# Root Traits, Nodulation and Root Distribution in Soil for Five Wild Lentil Species and *Lens culinaris* (Medik.) Grown under Well-Watered Conditions

**DOI:** 10.3389/fpls.2017.01632

**Published:** 2017-09-25

**Authors:** Linda Y. Gorim, Albert Vandenberg

**Affiliations:** Department of Plant Sciences, College of Agriculture and Bioresources, University of Saskatchewan, Saskatoon SK, Canada

**Keywords:** wild lentil species, root traits, root distribution, nodules, soil horizons

## Abstract

The efficient use of resources such as water and nutrients by plants is increasingly important as the world population food demand continues to grow. With the increased production of lentil in the temperate zones of North America, improvement in yield needs to be maintained. The use of wild lentil genotypes as sources of genetic diversity for introgression into cultivated lentil is an important breeding strategy, but little is known about their root systems. We evaluated the root systems of five wild lentil species and *Lens culinaris* under fully watered conditions. Plants were grown in 60 cm tubes containing equal volumes of soil collected from the reconstructed A, B, and C horizons. Significant differences were observed for root traits and fine root distribution between and within species and the proportion of root biomass partitioned into each soil layer was unique for each genotype. We also observed variability in nodule number and nodule shape within and between genotypes. Some genotypes more efficiently used water for either biomass or seed production. The allocation of resources to seed production also varied between genotypes. These observations could have impact on the design of future lentil breeding in the context of strategies for managing changes in rainfall amount and distribution for lentil production ecosystems.

## Introduction

*Lens culinaris* evolved and was domesticated in Southwest Asia (Ladizinsky, 1993) from where lentil cultivation subsequently spread to suitable ecosystems in all continents. Because lentils cook quickly, global consumption is rising faster than human population growth, and production is increasing rapidly, especially in Australia and the northern plains of North America. As the world population continues to grow and competition for agricultural resources increases, there is a need to produce high-yielding lentil genotypes that efficiently use resources even under well-watered conditions. The efficient use of water and nutrient resource will become more important in the future as conflict increase for these resources (Sauchyn and Kulshreshtha, 2008; Dai, 2011). The world total fertilizer (N,P,K) use forecast for 2017 is 197 Mt thousand tons with 30 Mt 1000 tons of N fertilizer forecasted for use in North America (Food and Agricultural Organization of the United Nations [FAO], 2015). Because of predicted water conflicts, unsustainable demands for fertilizer in the long run and release of greenhouse gasses into the atmosphere, it is necessary to improve both water capture and biological nitrogen fixation. Breeding high yielding lentil cultivars that minimize water use and have the most efficient nitrogen use will be increasingly important. Future lentil cultivars must possess both effective root system for capturing moisture, while maximizing their nitrogen fixation. Some lentil cultivars grown in North America show variable nodulation ability (Abi-Ghanem et al., 2011). Among the *Lens culinaris* cultivars investigated, *L. culinaris* Eston exhibited better nodulation and the highest nodule activity compared to others. Ability to exploit this variation is part of a strategy to achieve maximum yield of lentil. Another part of this strategy is the selection for improvements in root traits and architecture. Roots systems affect nutrient and water uptake, and function as sinks for photoassimilates and carbon inputs into the soil (Gan et al., 2009). Although root systems vary from plant to plant in response to environment and due to genetic variation, the presence of very fine roots (diameter < 0.5 mm) and/or fine roots (diameter 0.5–2.0 mm) is an important physiological component that determines most of the root length and root surface area available for water and nutrient uptake (Zobel et al., 2007; Zobel and Waisel, 2010). However, the efficiency of water extraction from soil depends on whether there is a stored water reserve in the soil, or whether the water supply is driven by in-season rainfall (Tron et al., 2015). Ideally, lentil varieties should possess both deep root systems and roots that can spread over large soil volumes in order to capture surface water.

The root system of cultivated lentil (*Lens culinaris* Medik) has been compared to those of oilseeds, other pulse crops and wheat (Gan et al., 2009, 2011; Liu et al., 2010). The distribution pattern of fine root traits such as root length, root surface area, root volume, and root diameter in cultivated lentil were different from those of other crops, and therefore could play a major role in water and nutrient uptake. Gahoonia et al. (2005) reported that two lentil varieties with differences in root length produced corresponding yield differences. Given the narrow genetic base of lentil, their wild relatives may be a possible source for genetic resources. Using wild relatives of lentil has been for improvement of diseases resistance (Ye et al., 2002; Muehlbauer et al., 2006; Tullu et al., 2006; Podder et al., 2013). For example, Tullu et al. (2006) demonstrated that recombinant inbred lines developed from a cross between the susceptible *Lens culinaris* Eston and a resistant germplasm line (PI 320937) had resistance to ascochyta blight. Podder et al. (2013) demonstrated through screening of accessions of both cultivated and wild lentil, that more than 70% of wild accessions had resistance to stemphylium blight, and many also had resistance to other diseases such as ascochyta blight and anthracnose. Given the great amount of variability to biotic stresses in wild lentil species, there may be great potential to improve both root systems and nitrogen fixing ability of cultivated lentil. Little is known about the root system of wild lentil species in comparison to cultivated lentil. A few studies that focus on above ground parameters such as yield and plant height have been reported for wild lentil germplasm (Pratap et al., 2014; Singh et al., 2014). A recent study on wild lentil germplasm in the field and indoors, showed the phylogenetic relationship between genotypes in the context of drought tolerance but root traits were not characterized (Singh et al., 2016). A baseline study of drought tolerance of wild lentil species showed that variation in drought avoidance exists between and within species (Gorim and Vandenberg, 2017). No comprehensive study exists for wild lentil root traits and root distribution patterns in different soil layers.

This study focused on the distribution pattern of root traits and nodulation at different soil depths in both wild and cultivated lentil genotypes. We hypothesized that wild lentil genotypes belonging to specific gene pools would have similar root traits and nodule distribution in the soil. Our objective was to determine if differences do exist for root traits and nodulation traits between and within wild and cultivated lentil genotypes and between their distribution patterns in the different soil horizons.

## Materials and Methods

### Soil Materials

The experiment was carried out in the controlled environment facility at the College of Agriculture and Bioresources at the University of Saskatchewan, Canada (lat. 52.133; long. -106.631). Detailed description of the soil type, soil collection site, and fertilizer applied were similar to that reported by Gorim and Vandenberg (2017). The soil was placed into 10 cm diameter × 60 cm length tubes that were divided into three 20-cm sub-sections corresponding to the depths of the A, B, and C horizons observed in the field. Therefore, soil in the A horizon or top soil, ranged in depth from zero to 20 cm, B horizon ranged from 20 to 40 cm and C horizon from 40 to 60 cm. The tubes were duct taped together and sealed at the bottom with fine mesh to allow for drainage and prevent soil loss. The top section of each tube was filled with 1.8 kg of soil from the A horizon to allow sufficient space for watering. The middle and bottom tube sections were filled with 2 kg of soil from the B and C horizons, respectively.

### Plant Materials

The wild lentil species/genotypes (abbreviation in parentheses) were: *L. orientalis* (*L. ori.*), IG 72643, *L. ori.* PI 572376, *Lens tomentosus* (*L. tom.*) IG 72805, *L. odemensis* (*L. ode.*) IG 72623, *L. lamottei* (*L. lam.*) IG 110813, *L. ervoides* (*L. erv.*) L-01-827A, *L. erv.* IG 72815. *Lens culinaris* (*L. cul*.) Eston was the only cultivated genotype included in this experiment since it has been successfully crossed with some of the wild species. According to Wong et al. (2015), *L. cul*., *L. ori*., and *L. tom*. comprise the primary gene pool; *L. ode*. and *L. lam*. are in the secondary gene pool, and *L. erv*. is in the tertiary gene pool. These three gene pools are genetically accessible for introgression of traits through interspecific hybridization.

Seeds of the wild and cultivated lentil genotypes were scarified, washed in bleach and then pre-germinated in a dark chamber at 22°C. Seedlings with radicle length greater than 2 cm were selected after 3 days and then transplanted into labeled tubes, and 6 g of rhizobia inoculum (*Rhizobium leguminosarum* biovar *viceae* strain 1435; Nodulator XL SCG, Becker Underwood, Canada) was added to the soil surface next to the seedling in each tube. The experiment was a complete randomized block design with 8 genotypes, two harvest times (during pod filling and at full maturity) and four replicates. The amount of water at 100% of field capacity (FC) for six random unplanted tubes was predetermined. The soil in the tubes was maintained at 80% FC throughout the experiment. The temperature was set to 21°C day/15°C night day length set at 16 h. Light intensity ranged from 308 to 392 μmol⋅m^-2^⋅s^-1^ depending on tube position and plant height. The light bulbs in the room T-5 fluorescent bulbs (# 835 Philips, ON, Canada) and LED light bars, 730 mm Far Red, (Fluence Bioengineering, Austin, TX, United States). Tube positions within each block were re-randomized at each weighing throughout the experiment to minimize light position effects.

### Parameters Evaluated

Half of the plants were harvested to evaluate growth parameters at 11 weeks after sowing (WAS) while the other half were at 13 WAS when mature seeds had been produced. Days to flowering was recorded as the date when more than 90% of the plants had an open flower. At harvest, plant height was measured as the distance from the soil level to the tip of the uppermost leaf. The number of pods and seeds per plant was counted at maturity. The above ground biomass was measured by placing the entire plant in labeled paper bags, and then oven dried at 70°C for 48 h prior to weighing. The duct tape that sealed the joints of the 3 tube sub-sections was removed, then a knife was used to cut and separate each tube into their respective A, B, and C horizons. The soil of each sub-section was then washed away gently on a sieve with 0.5 mm mesh size to minimize root loss. The washed roots were placed in labeled Ziploc bags, and debris and dead roots from the soil were manually removed. Root traits were measured using commercial software WinRHIZO^TM^ (Regent Instruments Inc., Canada, 2013) to determined total root length (TRL), total root surface area (TRSA), root length per unit volume of soil [referred to as root length density (RLD)], total root volume (TRV), the mean root diameter (MRD), root volume (RV), the total number of root tips (TRT) and the total number of root forks (TRF) in each horizon. WinRHIZO^TM^ also generated additional output that categorize root traits into six diameter classes: 0 – 0.5 mm, >0.5 – 1 mm, >1 – 1.5 mm, >1.5 – 2.0 mm, >2 – 3.5 mm, and >3.5 – 4.5 mm. The proportion of roots in each class was calculated as percentage of the total for each of the three root tube sections.

Before root analysis, the number of nodules in each horizon of the soil for each species/genotype was counted and photographed. After WinRHIZO^TM^ analysis, the roots from each horizon were placed in labeled paper bags, oven dried at 70°C for 48 h and then weighed to obtain root dry weight. The set of plants that continued to grow were enclosed in fine mesh bags to prevent shattering loss and harvested when greater than 80% of seeds were dried on each plant. The number of seeds, seed weight and thousand grain weight were determined for each species/genotype.

### Statistical Analysis

The SAS 9.4 PROC GLM procedure (in Statistical Analysis System, SAS Institute, Cary, NC, United States) was used to compare means among species/genotypes at given soil depths for each overall root trait and root dry weight. Letters were assigned to show significant differences at α = 5%. Least significant differences (LSDs) were also generated to compare species/genotypes belonging to a given diameter class at a given soil depth with the aid of SAS.

## Results

### Above Ground Growth Characteristics of *Lens* Genotypes

Above ground growth parameters for both cultivated and wild lentil were variable, having no bearing on their gene pool classification (**Table 1**). Variable plant height and flowering dates were observed among and between genotypes. *Lens orientalis* PI 572376 and *L. erv*. IG 72815 had the shortest and tallest plants respectively; the other genotypes had similar height. Most genotypes flowered between 36 and 42 days. The two *L. erv.* genotypes (tertiary gene pool) took the least and most days to flower. The total (root plus shoot plus seed) amount of biomass produced also varied amongst genotypes. The highest amount was produced by *L. cul*. Eston while the least was produced by *L. erv*. L-01-827A. At first harvest, *L. tom*. IG 72805 had the highest number of pods followed by *L. erv*. L-01-827A while both *L. ode*. IG 72623 and *L. erv.* IG 72815 had the least number of pods (**Table 1**). At the end of the experiment, the number of seeds produced and seed weight, varied among genotypes and their gene pools. *Lens culinaris* Eston produced significantly fewer but larger seeds compared to all the wild lentil genotypes (**Table 1**).

**Table 1:** Comparison of above ground phenotypic traits for *Lens culinaris* Eston and five wild lentil species grown under fully watered conditions (mean ± SE).

*Lens* species and genotype	Plant height (cm)	DTF (> 90%)	Total biomass (g)	No. of pods	No. of seeds	Seed yield (g/plant)	Thousand seed weight (g)
*culinaris* Eston	42.9 (±3.4)	42	18.4 (±1.9)	203 (±15)	205 (±9)	7.2 (±0.3)	35.0 (±0.4)
*orientalis* PI 572376	25.0 (±3.7)	42	13.8 (±0.5)	189 (±42)	629 (±27)	4.9 (±0.2)	7.7 (±0.1)
*orientalis* IG 72643	41.1 (±4.9)	36	12.7 (±1.1)	173 (±27)	277 (±31)	4.6 (±0.4)	16.5 (±0.5)
*tomentosus* IG 72805	40.3 (±4.6)	36	10.5 (±0.9)	361 (±33)	353 (±18)	4.6 (±0.2)	13.0 (±0.4)
*odemensis* IG 72623	43.8 (±6.2)	42	13.8 (±0.8)	61 (±11)	213 (±10)	3.3 (±0.1)	15.7 (±0.5)
*lamottei* IG 110813	39.2 (±6.9)	36	9.7 (±0.6)	179 (±24)	157 (±4)	2.5 (±0.7)	15.6 (±0.2)
*ervoides* L-01-827A	39.8 (±5.7)	28	8.1 (±1.4)	240 (±33)	551 (±101)	3.6 (±0.6)	6.6 (±0.1)
*ervoides* IG 72815	46.2 (±1.1)	60	10.6 (±0.5)	41 (±14)	264 (±27)	2.0 (±0.2)	7.4 (±0.2)


### Comparisons of Biomass Production of Wild and Cultivated Lentil

**FIGURE 1:**
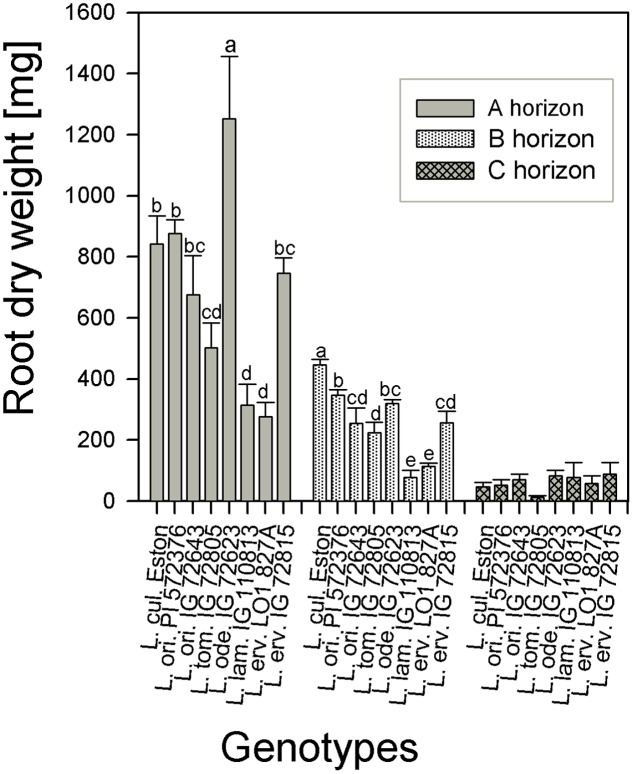
Comparison of root dry weight distribution in three soil horizons for five wild lentil genotypes and *Lens culinaris* Eston. [Bar are standard errors and letters compare root dry weight between genotypes for the same soil horizon; no letters imply similar biomass].

**FIGURE 2:**
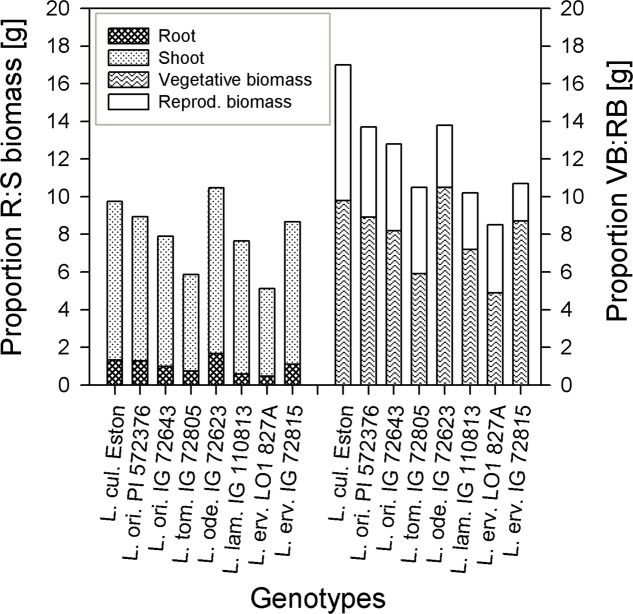
Comparing proportions of root (R) to shoot (S) biomass and vegetative biomass (VB) to reproductive biomass (RB) in five wild lentil species to *Lens culinaris* Eston.

The amount of root biomass produced in the three soil horizons was different within and between genotypes (**Figure 1**). In the A horizon, *L. ode.* IG 72623 produced significantly more (α = 5%) root biomass compared to all other genotypes. Genotypes belonging to the primary gene pool had similar root biomass except for *L. tom.* IG 72805 which had significantly lower biomass. *Lens lamottei* IG 110813 and *L. erv.* L-01-827A belonging to the secondary and tertiary gene pool respectively, both had significantly lower (α = 5%) root biomass compared to all other genotypes in both the A and B horizons. *Lens culinaris* Eston, however, produced significantly higher biomass compared to all other genotypes in the B horizon. For all genotypes investigated, the least amount of root biomass was observed in the C horizon, and there were no significant differences among genotypes at this depth (**Figure 1**). For all species, when the amount of root to shoot produced was compared, it was observed that all lentil genotypes allocated more of their resources toward shoots. The highest vegetative biomass (significant at α = 5%), was produced by *L. ode.* IG 72623 although this did not translate to a corresponding increase in reproductive biomass. *Lens culinaris* Eston produced the highest amounts of vegetative and reproductive biomass combined (**Figure 2**).

### Comparison of Root Traits between Wild and Cultivated Lentil

**FIGURE 3:**
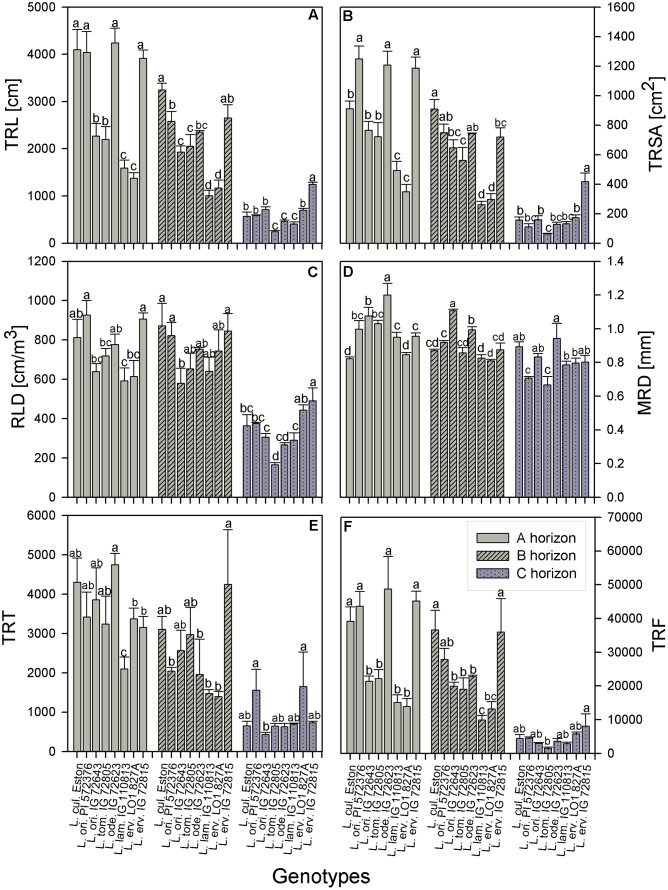
Comparison of root trait characteristics in three soil horizons for *Lens culinaris* Eston and five wild lentil species grown under fully watered conditions. Bars denote standard error. Letters denote significant differences at α = 5% for genotypes in the same soil horizon. **(A)** TRL, total root length; **(B)** TRSA, total root surface area; **(C)** RLD, root length density; **(D)** MRD, mean root diameter; **(E)** TRT, total number of root tips; **(F)** TRF, total number of root forks.

**FIGURE 4:**
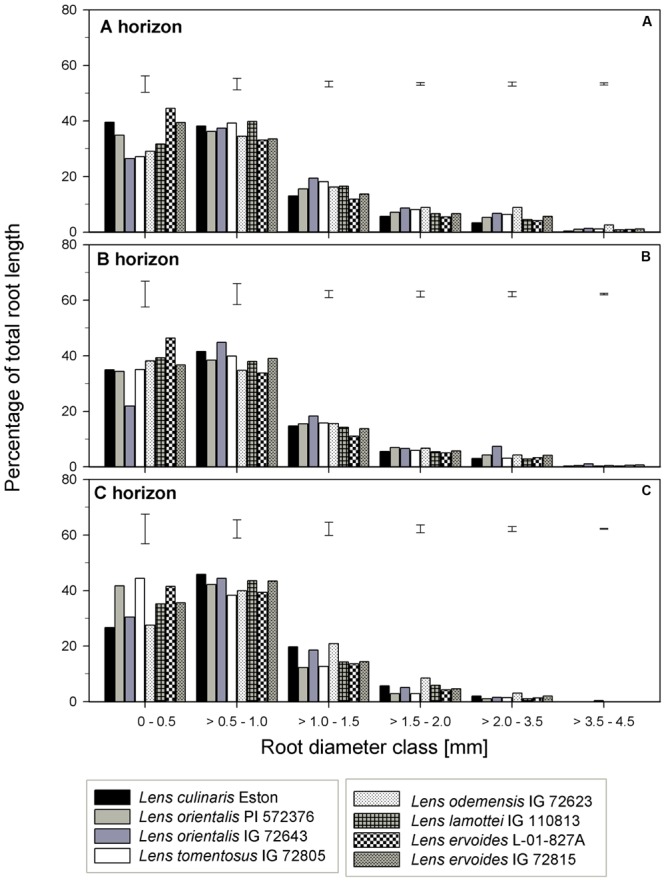
Percentage of total root length distribution across six root diameter classes assessed in three soil horizons for seven wild lentil genotypes in comparison to cultivated lentil (*Lens culinaris* Eston. The line bars are LSD values at *P* < 0.05 for comparison of genotypes within a diameter class. **(A)** A horizon; **(B)** B horizon; **(C)** C horizon.

TRL, RLD, TRSA, MRD, TRT, and TRF were analyzed in all soil horizons to gain understanding of how root growth differed among genotypes/species. We further assessed the fine root distribution patterns in each soil horizon to determine if root traits varied among genotypes and gene pools. We report root trait results in terms of gene pool classification and soil horizons. In the A horizon, *L. cul*. Eston, *L. ori*. PI 572376, *L. ode*. IG 72623, and *L. erv*. IG 72815 had significantly high (α = 5%) TRL although they represented three different gene pools (**Figure 3A**). *Lens orientalis* IG 72643 and *L. tom*. IG 72805, both primary gene pool species, had intermediate TRL, but this was significantly higher (*P* < 0.001) compared to that of *L. lam*. IG 110813 and *L. erv.* L-01-827A (secondary and tertiary gene pool, respectively). *Lens culinaris* Eston, *L. ori*. PI 572376 and both *L. erv.* genotypes had significantly higher (α = 5%) percentages of roots in the 0 – 0.5 mm diameter class. At root diameters > 1.5 mm, the percentage of TRL in *L. cul*. Eston in most cases was significantly lower than those of wild lentils (**Figure 4A**). *Lens ervoides* L-01-827A had a consistently high proportion of its TRL in the diameter class, 0 – 0.5 mm.

**FIGURE 5:**
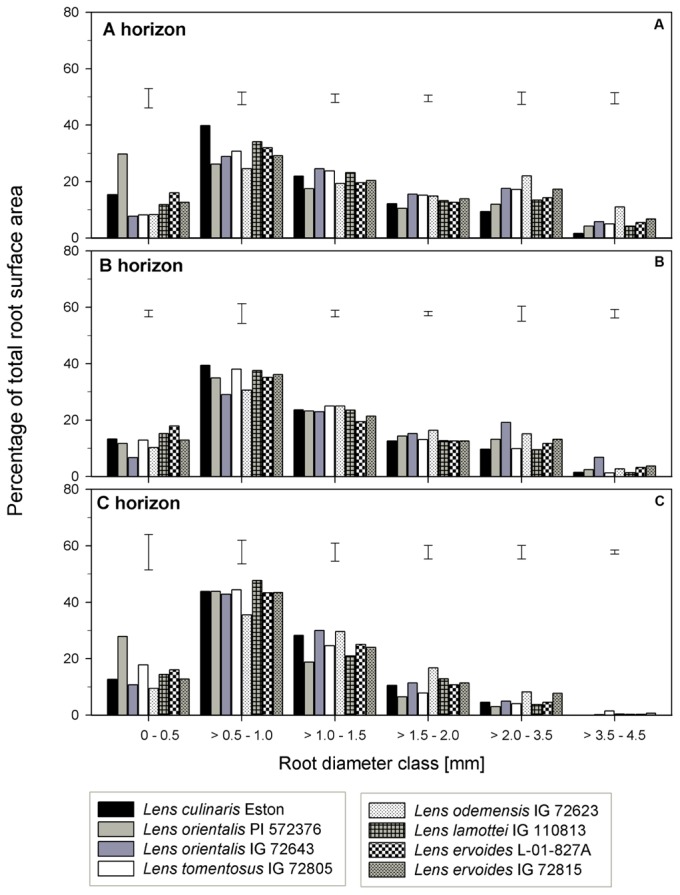
Percentage of total root surface area distribution for six root diameter classes in three soil horizons for seven wild lentil genotypes in comparison to cultivated lentil (*Lens culinaris* Eston). The line bars are LSD values at *P* < 0.05 for comparison of genotypes within a diameter class. **(A)** A horizon; **(B)** B horizon; **(C)** C horizon.

The TRSA was significantly higher for *L. ori*. PI 572376, *L. ode*. IG 72623 and *L. erv*. IG 72815, although they belong to the primary, secondary and tertiary gene pools, respectively. *Lens lamottei* IG 110813 and *L. erv*. L-01-827A had the lowest TRSA (significant at α = 5%). When the proportions of TRSA were classified into different diameter classes, significant variations in how individual genotypes partitioned their TRSA was observed (**Figure 5**). *Lens orientalis* PI 572375 had a significantly higher proportion of TRSA in the root diameter class of 0 – 0.5 mm compared to all other genotypes (**Figure 5A**). Most species had their highest proportion of TRSA in the diameter class > 0.5 – 1.0 mm but within this class, the TRSA of *L. cul*. Eston was significantly higher (α = 5%) than those of all wild lentils. *Lens odemensis* IG 72623 had the highest (significant, α = 5%) proportion of TRSA in the diameter class > 2.0 – 4.5 mm (**Figure 5A**) compared to the other genotypes. *L. tom*. IG 72805, had a higher proportion of its TRSA in the root diameter class > 2.0 – 4.5 mm in this horizon compared to the other soil horizons. MRD was variable between and within genotypes in all soil horizons, no trends were observed that could be linked to gene pool categorization (**Figure 3D**). The MRD of *L. cul.* Eston and *L. erv*. L-01-827A were similar, but significantly lower (α = 5%) compared to all other genotypes. *Lens odemensis* IG 72623 had the highest (significant, α = 5%) MRD.

**FIGURE 6:**
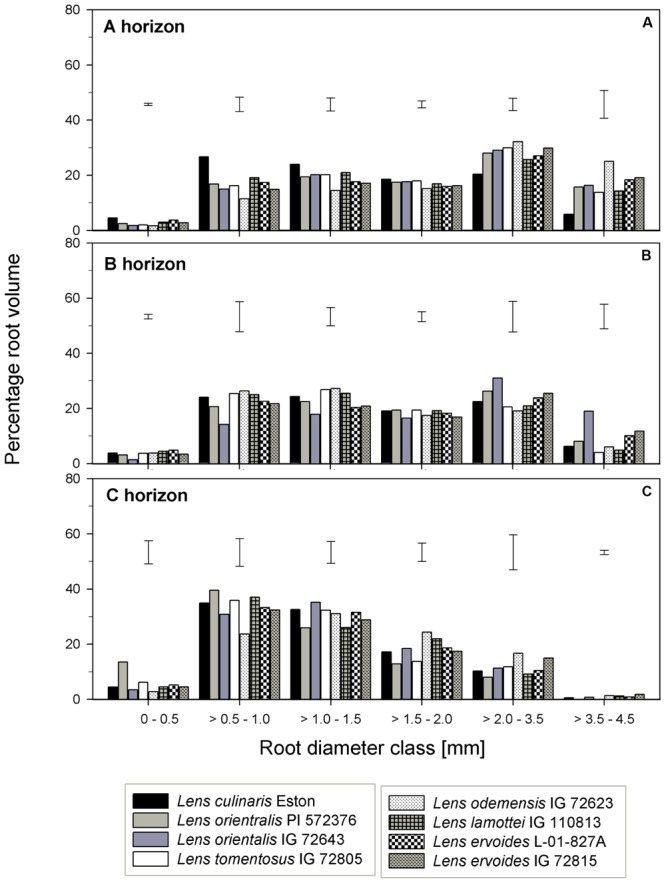
Root volume distribution across six diameter classes assessed in three soil horizons in five wild lentil genotypes in comparison with cultivated lentil (*Lens culinaris* Eston). The line bars represent LSD at *P* < 0.05 for comparison within a given root diameter class. **(A)** A horizon; **(B)** B horizon; **(C)** C horizon.

For genotypes in the primary gene pool, *L. cul*. Eston had the highest proportion of RV in root diameter classes < 2 mm and the least in root diameter classes > 2 mm (**Figure 6A**). For genotypes in the secondary gene pool, *L. ode*. IG 72623 had significantly lower (α = 5%) proportion of roots in root diameter classes < 2 mm compared to *L. lam*. IG 110813, but had a higher proportion of RV at root diameters classes > 2 mm. Genotypes in tertiary gene pool had similar RV in most diameter classes (**Figure 6A**). The TRT also varied between and within genotypes, but no clear patterns emerged because of high variability. *Lens lamottei* IG 110813 had the lowest TRT (significant, α = 5%) TRT, while that of *L. cul*. Eston was similar to those of the other species (**Figure 3E**). The TRF was significantly higher (α = 5%) for *L. cul*. Eston, *L. ori*. PI 572376, *L. ode*. IG 72623 and *L. erv*. IG 72815 compared to the other genotypes.

In the B horizon, *L. cul.* Eston had the significantly higher (α = 5%) TRL than all other genotypes except that of *L. erv*. IG 72815. TRL varied significantly among wild lentil species. The lowest TRL (α = 5%) was observed in *L. lam*. IG 110813 and *L. erv*. L-01-827A. *Lens ervoides* L-01-827A had a high proportion of its TRL in the diameter class, 0 – 0.5 mm compared to all the other diameter classes. On the other hand, *L. ori*. IG 72643 which had significantly low proportions of TRL in the diameter class 0 – 0.5 mm and significantly higher proportions of TRL in diameter classes > 0.5 mm (**Figure 4B**). *Lens culinaris* Eston had an increased proportion of roots in the diameter class, 0.5 – 1.0 mm. At root diameter class > 1.0 mm, *L. ori*. IG 72643 had significantly higher (α = 5%) proportions of TRL compared with *L. cul*. Eston.

*Lens lamottei* IG 110813 and *L. erv*. L-01-827A had similar, but significantly lower (α = 5%) TRSA compared with all other genotypes (**Figure 3B**). *Lens culinaris* Eston had similar TRSA compared to *L. ori.* PI 572376, and *L. ode*. IG 72623. All three had significantly higher (α = 5%) TRSA than the other genotypes. When the TRSA distribution pattern into different diameter classes was assessed, all genotypes had the highest proportion of their roots in the diameter class > 0.5 – 1.0 mm. *Lens orientalis* IG 72643 had significantly higher (α = 5%) TRSA proportions compared to all other genotypes in the root diameter class > 2.0 – 4.5 mm (**Figure 5B**).

*Lens orientalis* IG 72643, followed by *L. ode*. IG 72623, had the highest MRD (significant at α = 5%) and both *L. lam*. IG 110813 and *L. erv*. L-01-827A had significantly lower (α = 5%) MRD than other species. The remaining species had MRD that were similar to those of *L. cul*. Eston (**Figure 3D**). Similar proportion of RV were observed in all genotypes at root diameter classes > 0.5 – 1.0 mm and >1.5 – 3.5 mm (**Figure 6B**). *Lens oriental* IG 72643 had a significantly lower (α = 5%) proportion of RV compared to the other genotypes at root diameter classes 0 – 0.5 and >1 – 1.5 mm. For diameter classes > 3.5 – 4.5 mm, *L. ori*. IG 72643 and both *L. erv*. genotypes had significantly higher (α = 5%) proportions of RV compared to the other genotypes (**Figure 6B**).

All wild lentils had similar TRT when compared to *L. cul*. Eston (**Figure 3E**). However, the TRF were similar in *L. cul*. Eston, *L. ori*. PI 572376 and *L. erv*. IG 72815, and these were significantly higher (α = 5%) than that in the other genotypes. *Lens lamottei* IG 110813 and *L. erv*. L-01-827A had similar TRF but this was significantly lower (α = 5%) compared to that of *L. cul*. Eston.

In the C horizon, TRL was similar for genotypes in the primary gene pool except for *L. tom*. IG 72805 which was significantly lower (α = 5%) than the rest. TRL values were similar for the genotypes in the secondary gene pool, and not different to that of *L. cul*. Eston. In the tertiary gene pool, however, *L. erv*. IG 72815 had significantly higher (α = 5%) TRL compared to *L. erv*. L-01-827A, also in comparison to all other genotypes. Although the distribution of TRL proportions varied between and within genotypes, the overall shape of the distribution into different root diameter classes was similar (**Figure 4C**). Most TRL proportions fell in the diameter class 0 – 1.0 mm and no distinct patterns were observed among genotypes belonging to different gene pool (**Figure 4C**). At diameter class 0 – 0.5 mm, *L. tom.* IG 72805 had TRL that was similar to that of *L. ori* PI 572376 and *L. erv*. L-01-827A, but significantly higher (α = 5%) than those of the other wild lentils including *L. cul*. Eston. In the > 0.5 – 1.0 mm root diameter class, *L. cul*. Eston had similar TRL to all genotypes except *L. tom*. IG 72805, which had significantly lower (α = 5%) TRL. *Lens culinaris* Eston, *L. ori.* IG 72643 and *L. ode*. IG 72623 had similar but significantly higher (α = 5%) proportions of TRL for root diameter class > 1.0 – 1.5 mm. All the wild lentil genotypes had similar proportions of TRL compared to *L. cul*. Eston at root diameter classes > 1.5 mm (**Figure 4C**).

*Lens tomentosus* IG 72805 and *L. erv*. IG 72815 had significantly (α = 5%) lower and higher t TRSA, respectively, compared to all other lentil genotypes (**Figure 3B**). At root diameter 0 – 0.5 mm, *L. ori*. PI 572375 had similar TRSA compared to *L. tom*. IG 72805, *L. lam*. IG 110813 and *L. erv*. L-01-827A and this proportion was significantly higher (α = 5%) than those of the remaining genotypes (**Figure 5C**). For the root diameter class, >0.5 – 1.0 mm, all wild genotypes had similar proportions of TRSA compared to *L. cul*. Eston. *Lens orientalis* PI 572376 and *L. lam*. IG 110813 had significantly lower (α = 5%) proportions of TRSA at root diameter class > 1.0 - 1.5 mm when compared with the other genotypes. *Lens odemensis* IG 72623 had significantly higher (α = 5%) proportions of TRSA in >1.5 to 2.0 mm compared to the other genotypes (**Figure 5C**).

The MRD of most wild genotypes were similar to those of *L. cul*. Eston except for *L. ori* PI 572376 and *L. tom*. IG 72805 (**Figure 3D**). In the C horizon, a high proportion of RV in all genotypes fell in the root diameter classes, 0.5 – 3.5 mm (**Figure 6C**). Although *L. ode*. IG 72623 had a higher proportion of RV at root diameter class at 0 – 0.5 mm diameter, this was not significantly different from that of *L. cul*. Eston. At all diameter classes, there were no significant differences in TRT and TRF between any of the wild lentils and *L. cul*. Eston (**Figures 3E,F**).

### Nodulation of Wild Lentils Compared with *L. culinaris*

All lentil genotypes were inoculated with the same strain of rhizobium but the soil used for this experiment was not sterilized. In the A horizon, *L. lam.* IG 110813 and *L. cul.* Eston were the genotypes with the lowest and highest mean nodule count, respectively, and these were significantly different (α = 5%) from those of the other wild lentils. In the B horizon, *L. erv.* L-01-827A had the lowest (α = 5%) mean nodule count. Overall, *L. cul*. Eston had the highest mean nodule count combined over the A and B horizons. No nodules were produced in the C horizon. There was a wide range in the number of nodules found between replications, therefore the total, mean and range of the total nodule count for the A and B horizons combined are presented by genotype (**Table 2**). All genotypes evaluated had more nodules in the A horizon compared to the B horizon. Among wild lentil genotypes, *L. tom*. IG 72805, *L. erv*. IG 72815 and *L. ode*. IG 72623 had the highest mean nodule counts that were significantly different (α = 5%) only from those of *L. lam*. IG 110813 and *L. erv*. L-01-827A.

**Table 2:** Range and mean of nodule number in the A and B soil horizons for five wild lentil species compared with *Lens culinaris* Eston (Mean ± SE).

Lens species and genotype	A horizon		B horizon		Total nodule count per species/genotype	
			
	Range	Nodule	Range	Nodule	Range	Mean
	Min Max	number	Min Max	number	Min Max	
*culinaris* Eston	270–766	482 (±108)	3–166	75 (±40)	273–807	539 (±127)
*orientalis* PI 572376	155–218	198 (±14)	21–42	31 (±4)	187–260	229 (±15)
*orientalis* IG 72643	64–109	91 (±12)	4–49	23 (±12)	68–149	114 (±21)
*tomentosus* IG 72805	110–709	369 (±130)	9–143	74 (±34)	119–829	443 (±147)
*odemensis* IG 72623	108–699	433 (±129)	15–39	25 (±6)	129–714	452 (±125)
*lamottei* IG 110813	33–55	46 (±6)	13–18	15 (±1)	46–68	60 (±6)
*ervoides* LO1 827A	15–100	60 (±19)	1–4	2 (±1)	19–101	62 (±18)
*ervoides* IG 72815	321–561	440 (±68)	7–92	33 (±20)	330–653	473 (±83)


**FIGURE 7:**
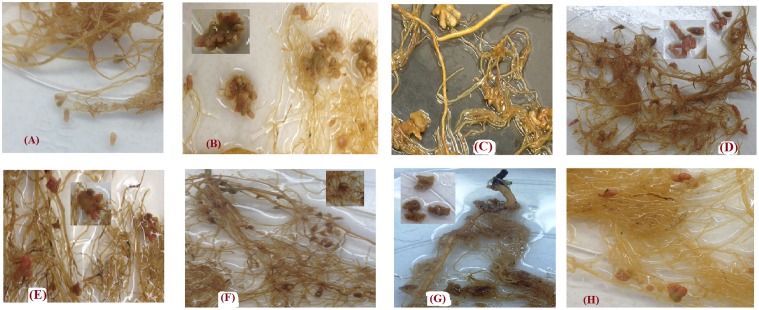
Variations in nodule shapes found in the root systems of *Lens culinaris* Eston and seven wild lentil genotypes inoculated with strains of *Rhizobium leguminarum* (*Rhizobium leguminosarum* biovar *viceae* strain 1435; Nodulator XL SCG, Becker Underwood, Canada). **(A)** *L. cul*. Eston **(B)** *L. orientalis* PI 572376 **(C)** *L. odemensis* IG 72623 **(D)** *L. lamottei* IG 110813 **(E)** *L. orientalis* IG 72643 **(F)** *L. tomentosus* IG 72805 **(G)** *L. ervoides* L-01-827A **(H)** *L. ervoides* IG 72815.

In most cases, the shape of nodules produced by each genotypes in this experiment was unique. *Lens culinaris* Eston developed long single nodules with rounded ends, similar in shape to nodules was observed in *L. tom*, IG 72805 (**Figures 7A,F**). The nodules of *L. ori*. PI 572376 formed attachments to roots as a single base which developed several buds, creating the shape of a cluster (**Figure 7B**). *Lens orientalis* IG 72643 nodules were attached to the root by a large palm-shaped surface with bifurcations resembling fingers (**Figure 7E**). *Lens odemensis* IG 72623 formed a massed nodule cluster with finger-like branches (**Figure 7C**). *Lens lamottei* IG 110813 had single long nodules with a green based an intense pink color toward their tips (**Figure 7D**). *Lens ervoides* L-01-827A had large nodules that were attached by a narrow base to the root but fanned out into finger-like buds (**Figure 7G**) while *L. erv*. IG 72815 had oblong nodules with green bases that appeared either singly or in pairs (**Figure 7H**).

## Discussion

Extensive differences between wild lentil genotypes and *L. cul.* Eston were observed for most of the parameters evaluated. Plant height was similar in all genotypes except for *L. ori*. PI 572376 which had significantly shorter plants. Although short, it ranked as one of the genotypes with a well-established root system extending into the C horizon. This may indicate that plant height is not a reliable indicator of rooting depth, and therefore not a predictor of drought tolerance for wild lentil genotypes, especially since most of the wild lentil species are described as prostrate in growth habit.

Most genotypes flowered at 36 – 42 days after sowing except for the 2 *L. ervoides* species, which flowered significantly earlier (*L. erv*. L-01-827A) or later (*L. erv*. IG 72815) than the others. This could reflect a possible gene by environment interaction in the centers of origin where these genotypes evolved (Ladizinsky, 1993) or a specific responses to indoor light quality (Yuan et al., 2017).

Wild lentil genotypes produced numerous light weight seeds that were small in comparison to compared to *L. cul.* Eston (see **Table 1**). The number of pods was not directly related to the total number of seeds produced given their indeterminate growth habit which resulted in continuous formation of flowers, pods and seeds. Small seeds may be an adaptive strategy that ensures seed dispersal in the wild but could potentially reduce yield when introgression to large-seeded *L. culinaris* is intended. Three of the wild genotypes (*L. ori*. PI 572376, *L. ode* IG 72623 and *L. erv*. IG 72815) had well developed root systems, but only a small proportion of their biomass was converted to seed yield (**Figure 2**). The effect was even greater under moisture deficit conditions (data not shown). However, more numerous and small seeds could be beneficial for adding for genetic diversity to increase yield in breeding programs in South East Asia where small-seeded *Lens culinaris* is preferred.

The root systems of cultivated lentil as well as other legumes grown under field conditions in Saskatchewan had been shown to be shallow with 77 – 85% of roots biomass found in the first 40 cm of soil (Gan et al., 2009). We found that greater than 90% of root biomass occurred in the top 40 cm of soil, higher proportion than the amount reported in the field. This could have resulted from the restrictions imposed by the sides of the tubes on root spread during growth, forcing lateral roots to grow downward. Having well developed deep root systems that extend into deeper soil layers is important as most agricultural systems become prone to drought spells. Root biomass was partitioned differently in the three soil depending on genotype studied (**Figure 1**). This implies that variability root biomass can be used to further improve cultivated lentil.

Specific root traits may also have a potential impact on future genetic improvement of lentil. Assessment of proportion of root distribution into different soil horizons (see **Figures 4**–**6**) showed that in the A horizon, similar TRL and RLD were observed for *L. cul*. Eston and wild genotypes such as *L. ori*. PI 572376, *L. ode*. IG 72623 and *L. erv*. IG 72815, each representing a different gene pool. However, when the proportions of roots distributed to the different root diameter classes was considered, no similarity was observed (**Figures 3A,C**). In the C horizon, most wild genotypes had higher TRL proportions at root diameters < 0.5 mm (i.e., more very fine roots) compared with *L. cul*. Eston implying that they were able to exploit both water and nutrients more efficiently given that the main functions of fine roots is water and nutrient uptake (Liu et al., 2010). TRL with root diameters > 2.0 mm were found in 2 wild lentil species (*L. ori*. IG 72643 and *L. ode.* IG 72623) in both the A and B horizons, which could imply they are more efficient in long distance transportation of water and nutrients to the shoot. These genotypes may be candidates for use in breeding strategies where simultaneous productivity under both moisture deficit and nutrient deficiency is required. Roots in the diameter class 0 – 2.0 mm, are known to have high plasticity and the potential to change their growth and development in order to adjust to changing environment, for example in rice in response to drought (Wang et al., 2009). This suggests there is wide scope for plasticity in both wild and cultivated lentil species since most of their TRL falls within this range in all three soil horizons. This could have implications for selection under drought conditions.

Gene pool categorization of both wild and cultivated lentil species had variable bearing on root traits. For example, *L. ori*. IG 72643 and *L. tom*. IG 72805 (primary gene pool) which had similar root biomass, TRL and RLD, also had similar TRL distribution in the A horizon across all diameter classes. This was not the case in the B horizon where *L. ori*. IG 72643 in comparison to *L. tom*. IG 72805, had significantly lower proportions of TRL in the 0 – 0.5 mm diameter class, implying reduced potential water and nutrient absorption. Furthermore, *L. lam*. IG 110813 and *L. erv*. L-01-827A (secondary and tertiary gene pools, respectively) had similar root biomass, TRL and RLD in the A and B horizons, but their TRL partitioning into various root diameter classes were different. *Lens ervoides* L-01-827A had most of its TRL in diameter classes < 1 mm while *L. lam*. IG 110813 had a higher proportions of TRL at diameter classes > 1 mm. The presence of very fine roots in all soil horizons, as observed in *L. erv*. L-01-827A could be the reason for its early flowering since it is able to absorb more moisture, and in the absence of thicker roots to store these moisture, channels the excess moisture into yield. This strategy could be exploited when early maturity is a trait of interest in breeding programs. Thicker roots (>2 mm diameter) with corresponding higher proportions of RV and TRSA have advantages in that thicker roots are able to penetrate hard soil surfaces. This allows them to forage more effectively for water and nutrients (Clarkson, 1985; Gu et al., 2017). These attributes were observed in both *L. ori*. IG 72643 and *L. ode*. IG 72623, making them prime candidates for introgression when breeding for environments that are prone to drought and nutrient scarcity.

Roots with larger diameters enable plants to penetrate compact soil thereby contributing to soil porosity (Goss, 1991) and larger pores promote the establishment of soil microbial communities (Lavelle et al., 2004). Below two mm diameter is considered the cut-off point that categorizes roots as “fine,” especially in other crops with larger root diameters (Merrill et al., 2005; Zobel, 2005). Liu et al. (2010) suggested there is a need to create a different diameter category scale for pulses, and for lentils in particular given that most of the roots of lentils fall in this diameter class. The assumption that these roots function the same way as the fine roots observed in cereals is not backed by any scientific assessments. However, the diameter scale reported in the present experiment differed from those reported in lentil field assessments by Liu et al. (2010). There were higher proportions of TRL and TRSA at root diameter classes > 2 mm for cultivated lentil in this experiments which was carried out indoors on the orthic dark brown chernozem, a similar soil as those used by Liu et al. (2010). Therefore, the differences in the proportions of root trait distribution may have resulted from different lentil growth conditions and the fact that different much wider genotypic range of lentil germplasm was assessed. Nonetheless, a root diameter scale needs to be established for each particular crop, lentil included, in order to reach meaningful conclusions with respect to root diameter classes.

Other important root traits include root volume (RV) and total root surface area (TRSA). RV is a mirror of the size of rooting systems, a key characteristic for plants to take up water and nutrients from the soil (Fageria, 2004). The RV is a product of root length and root diameter while the TRSA incorporates the root diameter. The root diameter influences the long-distance pathways for transporting water and nutrients to plant tissues, and makes up most of the TRSA responsible for exchange between root and soil (Waisel and Eshel, 2002; Gu et al., 2017). In the A horizon, *L. cul*. Eston and *L. erv*. L-01-827A had the smallest MRD while both *L. ori.* IG 72643 and *L. ode*. IG 72623 had significantly (α = 5%) higher MRD compared to that of L. *cul*. Eston in both the A and B horizons. Therefore, based on literature (Waisel and Eshel, 2002; Gan et al., 2011), both *L. ori.* IG 72643 and *L. ode*. IG 72623 roots with thicker diameter could facilitate long distance water and nutrient transport and storage compared to the other wild lentils and *L. cul*. Eston. It can be misleading to consider MRD alone during assessments given that it does not indicate which root diameter classes are present or their distribution in each genotype and soil horizon. It is important that the proportions of roots in different diameter classes be known to deduce the potential role and function of roots. For example, in the A and C horizons, *L. ori*. PI 572376 had most its TRSA in the diameter class of 0 – 0.5 mm (**Figure 4**). This significantly higher (α = 5%) TRSA proportion may have enabled it to acquire more nutrients and water, leading to the high number of seeds it produced. It could be useful to understand the response this species employs under drought conditions.

Root volume also plays a role in nutrient uptake. Previous studies in the field by Gan et al. (2011) reported that *L. culinaris* ‘CDC Glamis’ accumulated RV of about 35 mm^3^cm^-3^ in the field at late flowering. Our indoor results showed that *L. cul*. Eston accumulated higher RV (55.4 ± 7.3 mm^3^cm^-3^), and also had different proportion of the RV in the three soil horizons. They reported that in lentil, 59.0, 20.8, and 16.2 percent RV was accumulated at soil depths of 0 – 20, 20 – 40, and 40 – 60 cm respectively, with the balance at deeper layers. We found 47.6, 42.9 and 9.5 percent RV at these soil depths. The increased proportion of RV in our study in the B horizon may have resulted from the loosening up of the soil surface during tube filling, which could enable more root access into deeper soil layers.

High variability was observed in the total number of root tips, which were mostly found in the root diameter class ≤ 1 mm (data not shown). This could result from the influence of root washing after separation of the three soil horizons. This could possibly led to increased numbers of root tips, which were indistinguishable from the baseline number of real tips detected by the “WinRHIZO^TM^” root analysis program. Since the introduced variability could not be estimated, differences in total root tips were reported as an indicator as the real values were shadowed. Nonetheless, root tip numbers do provide insight on how roots penetrate into and proliferate in the soil layers. The values reported may serve as a guide in future studies of root sensory ability and synthesis of plant growth regulators. The same reasoning applies to an explanation for the variability observed in the total number of forks.

Roots also act as the site of nitrogen fixation. The amount of nitrogen fixed in legumes is a combination of how much the plant can remove from the soil and how much is fixed by rhizobia in their nodules. Lentil has been reported to have preference for biological nitrogen fixation (Zakeri et al., 2012). In this study, soil was depleted of nitrogen and none was applied. Therefore, almost all nitrogen in the dry matter produced is assumed to have resulted from nodule activity. We found that the number of nodules formed on the roots of wild and cultivated lentil in the A and B horizons was highly variable. This variability may have resulted from nodulation with native bacteria from the soil as well as those from the inoculum applied or both. *Lens culinaris* Eston had a far higher number of nodules compared to that reported by Abi-Ghanem et al. (2011). A possible reason for this may be the differences in rhizobia strains that were employed coupled with the short duration of their experiment. *Lens culinaris* Eston had the highest number of nodules, but when its total biomass (both vegetative and reproductive) produced was divided by the total nodule number, *L. cul*. Eston was not as efficient at fixing nitrogen in comparison to *L. ori*. IG 72643, *L. lam*. IG 110813, and *L. erv*. L-01-827A. This was further demonstrated by relatively low SPAD values of cultivated lentil compared to wild lentil genotypes (data not shown). Therefore, there is a need to further improve the nitrogen fixing capacity of cultivated lentil, and the use of wild lentil species may provide useful genetic variation in this regard. Different genotypes had nodules with different shapes, and sometimes two different shapes were observed in the same plant (**Figure 7**). This raises several questions such as whether or not the optimum rhizobia strains may be species or genotype specific given that our soil was not sterilized. Furthermore, some nodules in wild lentil genotypes were transient. Why they were transient and what strains of rhizobia (from soil or inoculum) formed these nodules, need to be further investigated; it may be the case that the nitrogen fixation metabolism of nodules with variable color may be different. Hence, it is necessary to conduct in depth studies on nodulation biology across these lentil species to determine to what extent the observed variation was dependent on strain × genotype interaction. There may be potential to improve the biological nitrogen fixation of cultivated lentil through interspecific hybridization with *L. culinaris* which has been successfully developed for four of the wild species reported here.

## Conclusion

The selection of genotypes for this study was made on the basis of their potential for hybridization with *Lens culinaris.* A wide range of phenotypic and genetic diversity exists in wild lentil genotypes for almost all traits related to above and below ground phenotypes. This diversity can be further studied and exploited as a means of improving the resistance of cultivated lentil to a wide range of biotic and abiotic stresses. Interspecific hybrids are available for crosses between *Lens culinaris* and the four wild species *L. orientalis, L. odemensis, L. tomentosus*, and *L. ervoides*. Gene pool classification based on genomic analysis does not explain the pattern of variation found in root phenotypes in this study. A great diversity in the root traits was observed across even the small spectrum of wild lentil genotypes, and there were also significant differences in root traits between genotypes belonging to the same wild species (*Lens ervoides* and *L. orientalis).* Significantly higher values for all root traits in all 3 soil horizons were found in three genotypes (*L. ori*. PI 572376, *L. ode*. IG 72623 and *L. erv*. IG 72815) compared to *L. cul.* Eston. It was observed that in both cultivated and wild lentils, the majority of their TRL fell in diameter classes that were less than 2 mm, the uptake range referred to a ‘fine roots.’ However, what this means in the context of pulse crops needs to be further defined, as all their roots cannot be fine roots as defined for other crops, particularly for cereal root systems. While root biomass and root traits such as TRL, TRSA and RV are important, further insights into rooting systems can only be obtained by looking at root distribution patterns at various soil depths. Furthermore, wild genotypes with deep root systems allocated their resources mostly toward biomass production and not seeds, implying that when interspecific hybridization and introgression become part of a long term breeding strategy for lentil, it will be necessary to develop appropriate selection strategies for simultaneous selection of yield and root traits. A wide range in nodule numbers and shapes exists among wild lentil genotypes that begs for further research in the context of nitrogen fixation and potential yield.

## Author Contributions

LG: Responsible for design, execution, analysis of experiments and preparation of manuscript with input from supervisor. Approved the final version presented and verified that all aspects of this work are correct. AV: Development of initial concepts for experimental works as part of grant proposals accepted by various research funding agencies. Supervised LG and revised manuscript for publication.

## Conflict of Interest Statement

The authors declare that the research was conducted in the absence of any commercial or financial relationships that could be construed as a potential conflict of interest.
